# ‘The High Five Club’: Social Relations and Perspectives on HIV-Related Stigma During an HIV Outbreak in West Virginia

**DOI:** 10.1007/s11013-022-09769-2

**Published:** 2022-02-23

**Authors:** Sarah G. Mars, Kimberly A. Koester, Jeff Ondocsin, Valerie Mars, Gerald Mars, Daniel Ciccarone

**Affiliations:** 1 Department of Family and Community Medicine, University of California, San Francisco, 500 Parnassus Avenue, MU3E, San Francisco, CA 94143 USA; 2 Center for AIDS Prevention Studies, University of California, San Francisco, 550 16th St, San Francisco, CA 94158 USA; 3 Department of Anthropology (Honorary), University College London, Gower St, London, WC1E 6BT UK

**Keywords:** Injecting drug use, HIV, Stigma, West Virginia, Cultural Theory

## Abstract

In the United States, HIV outbreaks are occurring in areas most affected by the opioid epidemic, including West Virginia (WV). Cultural Theory contends that multiple cultures co-exist within societies distinguished by their differing intensities of rules or norms of behavior (‘grid’) or degree of group allegiance/individual autonomy (‘group’). Accordingly, we would expect that perceptions about HIV, including stigma, correspond with individuals’ grid/group attributes. To explore this, we conducted qualitative interviews with people who inject drugs (PWID) recruited from a WV syringe service program. This paper focuses on our unexpected findings on stigma during a coinciding HIV outbreak. PWID living homeless identified as belonging to a ‘street family’. Its members were mutually distrustful and constrained by poverty and drug dependence but despite their conflicts, reported openness between each other about HIV + status. Interviewees living with HIV perceived little enacted stigma from peers since the local outbreak. Contrasting stigmatizing attitudes were attributed to the town’s mainstream society. The ‘High Five’ (Hi-V) Club, expressing defiance towards stigmatizing behavior outside the street family, epitomized the tensions between a desire for solidary and mutual support and a fatalistic tendency towards division and distrust. Fatalism may hinder cooperation, solidarity and HIV prevention but may explain perceived reductions in stigma.

## Introduction

HIV-related stigma has been a concern since early in the epidemic’s history, not only because it involves social suffering and discrimination but also because it can deter people from seeking HIV testing, treatment and other help, increasing risk of further viral transmission (Jeffries et al. 2013; Guarino and Teper 2009). Efforts have been made to reduce stigma in health care settings and the wider community with varying success (Grossman and Stangl 2013). More recently, pharmacological developments reducing the risk of HIV transmission and offering the potential for ultimately stemming new transmissions entirely (UNAIDS et al. 2010), may alter stigma in the long term. However, after a period of stabilization or decline, US national HIV incidence has started to rise among people who inject drugs (PWID) (Centers for Disease Control and Prevention 2018). Recent outbreaks of HIV have been discovered among PWID in several US states (Peters et al. 2016; Evans et al. 2018; Wheeling-Ohio West Virginia Health Department 2018; Centers for Disease Control and Prevention 2018).

Since the late 1990s, the ongoing US opioid epidemic has greatly expanded the population of people who use and inject opioids (Midgette et al. 2019). Heroin, often mixed with or replaced by fentanyl, has spread to parts of the country previously unaffected. Fentanyl is a synthetic opioid 30–40 times more potent than heroin (Ciccarone 2017), with a faster onset and a shorter half-life. Many fentanyl analogs of varying potency are also circulating in the drug supply (Suzuki 2017). More recently there has been a substantial rise in methamphetamine use among people who use opioids (Glick et al. 2018; Strickland et al. 2019). Methamphetamine use presents both heightened behavioral and physiological risks for HIV transmission (Fulcher et al. 1999; Patterson et al. 2005; Spindler et al. 2007).

Pharmacological developments in HIV prevention and treatment have radically changed the landscape of risk in the US with both anti-retroviral treatment (ART) and pre-exposure prophylaxis (PrEP). In 2011 it was shown that ART could prevent sexual transmission of HIV by the reduction of viral load to very low levels and the year before, PrEP, a daily dose of anti-retroviral drugs, was found effective in protecting against the acquisition of new infection despite sexual exposure to HIV (Cohen et al. 2011; Grant et al. 2010). However, these benefits have not been distributed equally either globally or within the US. PWID are under-represented among risk groups receiving pre-exposure prophylaxis (Garner et al. 2018; Coleman and McLean 2016) and viral suppression levels among people living with HIV (PLWH) in the US (54%) lag behind other economically comparable countries (Kaiser 2019).

With the highest drug overdose rate in the US and experiencing recent HIV outbreaks, West Virginia is an important locus for the interaction of opioid use and HIV risk. It has the highest death rate from opioid-related overdoses (often involving fentanyl) in the US with an age-adjusted fatality rate of 42.4 per 100,000 in 2018 (Scholl et al. 2019). The state was categorized as at high risk for HIV in 2018 and has since suffered several outbreaks among people who inject drugs (Evans et al. 2018; CDC/amfAR 2018). While syringe service provision has expanded overall, harm reduction efforts remain highly controversial in the state (Ondocsin et al. 2020).

### Stigma

Erving Goffman’s original study, *Stigma: Notes on the Management of Spoiled Identity* (1963)*,* conceptualized stigma as a relationship rather than a fixed attribute, one that could change depending on context and that could be resisted (Goffman 1963). Misinterpretation or misappropriation of Goffman’s work has led to focus on stigma as a relatively static ‘negative attribute’ and to overly individualized analyses rather than viewing it as a social process (Parker and Aggleton 2003). In an attempt to reassert structural perspectives, Parker and Aggleton define the process of stigmatization as the ‘production of negatively valued difference’ (Parker and Aggleton 2003). In harmony with this intention, this paper uses ‘Cultural Theory’ to place stigma within social contexts as a dynamic relationship between different groups and individuals (Douglas and Wildavsky 1982).

Further refining the concept of stigma, Scramble, in his work on epilepsy, distinguishes between ‘enacted’ stigma, which describes actual discrimination or experiences of unacceptability and ‘felt’ stigma, which is the fear of such discrimination or reactions whether or not they actually occur (Scrambler 1998). Enacted stigma may be related to a perceived threat (Goffman 1963), and among men who have sex with men (MSM), a reduced fear of HIV transmission arising from using PrEP seems to have had a destigmatizing effect on MSM who were living with HIV (Koester et al. 2018). However, stigma and fear are not always so closely aligned as is the case with visible or evident disabilities (Goffman 1963). Whether enacted HIV-related stigma and fear of HIV are related among PWID is unclear.

### Cultural Theory

Understanding what underlies the wide range of behaviors and beliefs that arise from what appear to be a shared set of facts or circumstances, such as the current COVID-19 pandemic, continues to stimulate research and debate eg (Rieger 2020; Xu and Cheng 2021). Explanations for such variability are often rooted in individual psychology or material conditions alone, ignoring cultural biases. Cultural Theory (CT) has linked values and beliefs to social relationships, and from these, has explained many forms of behavior, both historical and contemporary (Douglas and Wildavsky 1982; Mars 2016; Weare, Lichterman, and Esparza 2014). Its application in studying stigma, both enacted and felt, offers the possibility of an understanding that goes beyond individual explanations.

Developed by anthropologist Mary Douglas and rooted in the writings of sociologist Emile Durkheim, CT contends that multiple cultures or ‘solidarities’ co-exist within societies (Douglas 1978; Durkheim 1951). These solidarities are distinguished by their differing intensities of rules or norms of behavior (‘grid’) and their degree of group allegiance or individual autonomy (‘group’). This approach is grounded in the belief that social relations generate preferences and perceptions that in turn sustain those relations in different cultural forms. Furthermore, the use of these two universally found cultural attributes (‘grid’ and ‘group’) allow comparisons to be made across diverse cultural contexts.

**Fig. 1:**
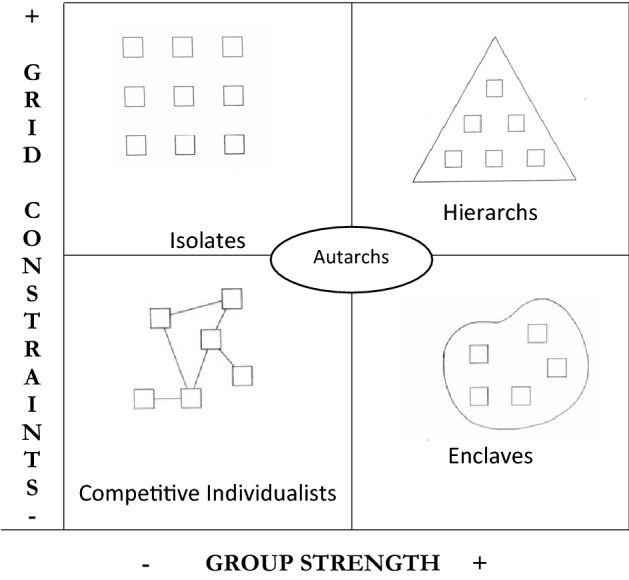
The five cultural theory archetypes. Adapted from Frosdick and Mars (Mars 2016) with permission (Frosdick and Mars 1999)

Cultural Theory solidarities exist in a state of flux and conflict with each other. The two dimensions of grid and group produce four archetypal solidarities not necessarily existing in these forms in real societies but useful for understanding the theory (see Fig. 1). Nor are these archetypes rigid or static as people can move between these categories depending on their context, circumstances and the changing appeal of what each offer (Thompson, Ellis, and Wildavsky 1990).

Low grid, low group ‘individualists’ or ‘opportunists’ make their own rules to compete effectively, network opportunistically with others and pursue short term goals for individual gain. They tend to be pragmatic in their approach to decision-making. An example would be a business entrepreneur such as a professional drug dealer who does not use drugs him or herself. High grid, low group ‘isolates’ or ‘fatalists’ are individuals controlled by rules and norms imposed from outside with limited ability to form solidarities with others. They have very little autonomy and, as a result, tend to be fatalistic about the future and their lives. An example would be a non-unionized parking lot attendant who works alone in a ticket payment booth according to a schedule determined by those in charge, though physical isolation from others is not a necessary requirement of isolates.

High grid, high group ‘hierarchs’ have highly structured groups with carefully delineated status, classification, many rules and clear lines of top down decision-making. They tend to take a long-term view beyond the lifespan of an individual, seeing the future and past in terms of the collective. An example would be the European feudal societies that built cathedrals over many generations. Low grid, high group ‘dissenting enclaves’ are groups that do not have many rules or fixed roles for members; decision-making may be democratic or may not follow strict structures; they maintain a strong boundary with the outside world, delineating those ‘inside’ versus ‘outside’. They also have a tendency to fragment. An example would be revolutionary socialist groups in which all members are equal. A fifth solidarity, introduced by Thompson, is the ‘autarch’ or hermit who tries to avoid social constraint by any group or imperative, valuing autonomy above all else. A self-employed gardener might be an autarch (Thompson et al. 1990).

Cultural Theory has been used to elucidate many different phenomena, including the selection of environmental risks and the successful and failed strategies of embattled medical factions (Douglas and Wildavsky 1982; Mars 2012). In the realm of HIV, Douglas and Calvez theorized that patterns of transmission risk perception and behavior correspond with individuals’ grid and group attributes (Douglas and Calvez 1990). However, no research is known of among PWID. The original aims of this research were to test Douglas and Calvez’ hypothesis of HIV risk perception among people who inject drugs in West Virginia. This will form the subject of a future publication. However, the research trip’s travel to West Virginia to collect data coincided with a local HIV outbreak, expanding the scope of the project and resulting in a remarkably rich dataset.

A striking finding was that, given how stigmatizing HIV has been as a disease since its identification, perceptions of felt and enacted stigma were very different: participants widely reported experiencing and observing less HIV-related stigma among their peers than prior to the outbreak. Enacted stigma was sometimes attributed to mainstream society and elicited a defiant response through the ‘Hi-V Club’.

This paper aims to place the understanding of perceived stigma in a cultural system. It argues that people who inject drugs and live homeless in this study show characteristics of ‘isolates’, their social relations are high grid and low group, constrained, controlled and divided by the short term demands of extreme poverty and drug dependence. This in turn produces a fatalistic outlook which hinders mutual trust and solidarity while also encouraging mutual tolerance and acceptance of HIV positive status. First, the paper describes the social relations and living conditions of those living homeless and using drugs in the city. We then turn to perceptions of stigma observed and experienced by the participants and consider both their own explanations for what they see as a decline in stigma since the HIV outbreak and those arising from Cultural Theory. Finally, we focus on the ‘Hi-V Club’ and the contradictory forces it exemplifies.

## Methods

This small study was made viable by nesting inside the larger National Institutes of Health funded Heroin in Transition study (PI: D. Ciccarone). Arrangements were made with a Syringe Service Program (SSP) in a town in West Virginia who agreed to host the two research projects for study recruitment. A team of interviewers, including JO and the two PIs, SGM and DC, traveled to West Virginia in September 2019 for one week of intensive qualitative research. For this project, 26 individuals were interviewed once each. Cultural characteristics have traditionally been assessed through ethnographic observation of a community. However, individual-level research has also been found to be a viable method of capturing cultural attributes, not by assessing culture directly but by exploring the products of relational processes (Rippl 2002), such as trust in authority and decision-making practices.

### Preparation

KK and SGM developed the initial interview guide and S carried out pilot interviews at Homeless Youth Alliance, one of San Francisco’s syringe service programs, to test interview questions and methods. After each pilot interview, SGM revised and refined the questions. The semi-structured interview guide was then revised for use in West Virginia, with questions focusing particularly on ‘group’, and allowing the ‘grid’ dimension to emerge through thick description of group. It was initially uncertain how well the San Francisco piloted interview guide would translate to the different local context of West Virginia but, through an iterative process, we refined the questions each morning of the interviews based on their reception and findings of the previous day. Most questions transferred well. Also arising from the pilot interviews was the phenomenon of the ‘street family’, a community of people living homeless and further explored in the West Virginia research.

The interview guide covered topics including who the interviewee considered ‘family’; whom they spent time with; trust and mutual reliance; beliefs around the causes of HIV transmission; personal opioid and blood borne viral risk perception and behavior; interaction between overdose prevention and HIV risk; PrEP awareness, preference and uptake; sexual behavior including transactional sex; changing patterns of drug use; community attachments/isolation and housing. Notes were taken after each interview about participants’ self-presentation and attire, partly to capture non-verbal modes of communication that would not be available on audio recordings and partly as a reminder of their identities in the subsequent data analysis process. All the interviews were transcribed in full.

#### Study Eligibility

Eligible participants had to be at least 18 years of age and self-reported as people who currently injected heroin as their primary drug, either living in or commuting to the research area. Exclusion criteria covered individuals who were intoxicated or otherwise unable to give informed consent or answer questions reliably. The study has ethical approval from University of California San Francisco’s Institutional Review Board.

#### Recruitment and sampling

As is appropriate with research on hidden populations, where the characteristics and dimensions of the whole population are unknown, purposive sampling was used (Barendregt et al. 2005). We intentionally aimed to recruit as near to equal numbers of men and women as possible since women are under-represented in research on illicit drug use (Tuchman 2010). With the agreement of the SSP staff, participants were recruited by the research team in the waiting room through a process of informed consent. SSP clients were approached partly on a ‘first-come-first served’ basis and partly based on a reading of their body language and facial expressions as to whether they looked open and approachable. Immediately upon recruitment, interviews were carried out in a private space or near the SSP and lasted about one hour. Participants were paid $20 for their time. The sum offered was agreed in consultation with the SSP staff who considered that, given the region’s economy and their past experience, a higher amount would result in an overwhelming response that would interfere with the SSP’s functioning.

At the recommendation of the UCSF Institutional Review Board, interviewers offered the participants the options of being quoted anonymously in publications and presentations or for the information to be used as background to the study and not quoted. Three of the 26 interviewees opted not to be quoted. All names have been changed in this paper to preserve confidentiality and no specific details provided that could identify interviewees but this option of an additional layer of anonymity may have enabled some interviewees to participate in the study who might otherwise have been uncomfortable sharing their thoughts and experiences.

#### Analysis

All interviews were professionally transcribed and each transcript treated as a unique research record. An analytic memo of significant observations was produced for each one based on the method utilized by Christopolous, et al. (Christopoulos et al. 2015): the semi-structured nature of the interview guides generated a memo structure for each interview to guide the analysis while avoiding forcing data into categories. Data corresponding with this structure was added to each memo from the transcripts. As new themes emerged, these were added to the memo structure, and transcripts were re-analyzed and the memos revised using inductive and deductive methods (Strauss and Corbin 1990). Negative cases that deviated from the bulk of the findings were also sought for comparison (Hamilton 2020) Thematic analytic memos summarizing findings across the interviews were also produced. The memos were compiled into a table to allow searching by theme or other content.

The transcripts and notes were simultaneously examined for evidence of Cultural Theory characteristics in participants’ attitudes and reported behavior. While it was not possible to classify each participant in terms of Cultural Theory based on a single interview, common themes arose across the body of the interviews suggesting trends towards particular solidarities.

## Results

### Geographical Context and Sample

West Virginia is a largely rural state with extremes of wealth and a population that is approximately 93% white (Spence et al. 2013; USCB 2020). Located in Appalachia, a region hard hit by de-industrialization and the opioid crisis, West Virginia is considered part of the MidWest and shares borders with Ohio, Kentucky, Virginia, Pennsylvania and Maryland.

We interviewed 26 people, of whom 11 were women and 15 were men. They ranged in age from their early 20s to their 50s. Eight mentioned that they were living with HIV, all diagnosed within the last year. Twenty-five people identified as white or Caucasian; one did not provide data. Most were living homeless or unstably housed. All but one, excluded from the analysis, were injecting heroin/fentanyl and many also used methamphetamine.

Experiences of intense social suffering suffused these interviews, including the huge death toll from opioid overdoses with the loss of friends and acquaintances. Some of the interviewees wept at the recollection of lost partners and friends, the trauma of their own overdose experiences or separation from their children. For those living homeless, daily life involved a constant round of finding money and drugs to keep opioid withdrawal at bay. Sex work was common among the women interviewed and mainly street based. One man mentioned that he exchanged sex for money also. Some of those interviewed saw little prospect of leaving current lives of illicit income generation, sex work and drug use despite a desire to do so, describing the paucity of legitimate economic prospects in the state.

#### The ‘Street Family’—Enclave or Fatalists?

Family allegiance was a frequent theme, as has been noted in West Virginia (Coyne et al. 2006), but separation from birth families/children was common. Many spoke of belonging instead to a ‘street family’ of local people living homeless and injecting drugs. Through the lens of Cultural Theory, the ‘street family’ would superficially appear to be a low grid, high group ‘enclave’ (see Fig. 1) with its language of group belonging, shared identity and desire for mutual cooperation. However, the context of drug dependence and near destitution had created conditions and beliefs that more clearly match those of isolated individuals or ‘fatalists’ (high grid, low group). A lack of trust and wariness was pervasive, intensifying suffering of street family members along with a sense of isolation although they were physically in the company of others. This paradox of being ‘together and alone’, was expressed by Jake, who was in his 40s and HIV negative:

I: […] Do you spend time with other people or are you mostly on your own?

S: I spend time with other people but I’m still on my own, if that makes any sense.

I: Yeah. You mean when you’re with them you’re on your own?

S: When I’m with them I’m on my own, that’s right. Because they’re drug addicts too and it’s dog eat dog.

Belonging to the ‘street family’ was not described as a desired choice or aspiration but as a necessity for those living homeless on the street, particularly for those who were single. Chris, living with HIV, explained:

We use each other to, uh, she [indicating another member of the street family] might have something I need. […] and I might have something she needs and in order to get money at the end of the day we gotta work together to accomplish what we need to do. That’s how our group works. […] You might not like each other, but this is how it is.

Those who were able to choose, through access to housing, reported separating themselves from the street family:

I: Okay. What about people that you're friends with now; your street friends. Do you consider them family?

L: Oh yeah. We look out for each other. We have to; it's survival.

I: So there's people around that you consider family and that you trust?

L: Well, I don't trust anybody.

I: Okay, so do you spend most of your time with other people or are you mostly on your own?

L:It really just depends on if I'm homeless or not. If I'm homeless, then I'm usually around a bunch of people. If I'm housed, I'm normally just in the house.

- Laurie, PLWH, in her 20s.

Andy, HIV negative and in his 40s, also expressed a preference for being alone but explained that homelessness prevented this: “I like being by myself, I’ve always been like by myself, but I’m always around people ’cause I am on the streets and the way to survive is kinda be around people and have each other’s back.”

Members of the street family relied on each other for the practical necessities of finding shelter, buying and sharing drugs and sharing information. They also used opioids together for fellowship and/or rescue during overdose. They encountered each other frequently on the streets. Yet while necessity and proximity pulled the street family together, strong forces divided it. Members frequently mentioned that the group failed to provide the emotional support or friendship they needed because of people stealing from each other and breaking trust. Jenni, a woman in her 40 s and HIV negative, expressed a typical view:

When you’re homeless, homeless steal from the homeless and then it’s like it don’t matter, you know […] Homeless steal from each other […] instead of uniting together and helping each other, it’s ridiculous. […] Everybody’s greedy, people, I mean, people are just out for theirselves.

Unlike a low grid enclave, poverty and drug dependence dominated decision-making, forming its own ‘grid’ of short-term imperatives and constraints that undermined the longer-term rhetorical and aspirational ideals of the group, intensifying their social suffering. Individuals were mutually distrustful and constrained while longing for cooperation. Sylvie, in her 40s, explained with passionate frustration that if only ‘all the kids that are out here on the streets with me’ put their energy into working together instead of planning to rob each other ‘not a fucking one of us would ever have to worry about being out in the cold in the middle of the winter. Not one of us would ever have to go without a fucking meal, you know, and not one of us would ever have to go dope sick.’ Improving the image of people using drugs within the town, such as by picking up litter or beautifying outside areas was also something Sylvie wished they could achieve together.

There didn’t appear to be any leadership to the group, perhaps reflecting the lack of trust between members, since without trust, a leader will not gain allegiance (Thompson et al. 1990). Rules were occasionally mentioned but these seemed to be ad hoc in their application and enforcement and no structure was evident. As Sylvie and Jenni’s words show, organizing to improve their conditions, reputation or access to resources was absent.

Although not everyone in the study could be said to have matched the characteristics of high grid and low group isolates, there was a strong tendency towards this solidarity, particularly among the street family. Fatalism, a cultural bias of isolates, tends towards a world view where change is brought about by external events that are inevitable and this emerged repeatedly in these narratives. Like other grid and group characteristics, fatalism does not have to be absolute, nor is it a psychological phenomenon but rather a way of explaining the world which in turn influences behavior. Yet some of the cultural characteristics common among isolates that made social relations so strained may also have played a part in the perceived reduction of HIV-related stigma.

#### Perceptions of HIV-Related Stigma

Many interviewees described a reduction in HIV-related stigma and greater acceptance of those who had been recently diagnosed with the virus in their circle compared to before the town’s outbreak, a view echoed in informal discussions with staff at the harm reduction program. Jenni saw openness and lack of stigma as a recent development, attributing this to the relative insignificance of HIV in the local context:

I: And are people pretty open about whether they have [HIV] or not?

S: Some are.

I: Some are, yeah, yeah. And do people treat them any different?

S: No.

I: No, interesting, yeah. Okay, do you think that’s always been so or just recently?

S: No, just recently.

I: Oh, why do you think that is?

S: I – cause it’s just such a outbreak, I mean, it’s – yeah. […] It’s like – it’s just another thing.

Her off-hand dismissal of HIV as ‘just another thing’ perhaps hints at the multiple adversities facing PWID in the area, that HIV in itself is not particularly significant. Among those who were living with HIV, several considered that among PWIDs, most were open about their HIV status, one man commenting ‘that ain’t nothing’ (Chris).

Opal, a woman in her fifties using heroin ‘off and on’ for over a decade, was asked the interview guide’s standard last question, “Any other questions that you think I should have asked about or anything else you wanna tell me?” to which she replied, “Well, I know that you haven’t asked this question, ‘Would I tell somebody if I had it, you know?’ And I would. I definitely would do that […]”.

Elsa, a sex worker in her 20 s, although not asked directly, volunteered the information that she had been diagnosed with HIV,

I: Um, so with your last date, um, did you know the person before or no?

S: No. […]

I: And did you use any kind of protection or –

S: Yeah, I always used a condom when I went.

I: Right. Um, and do your clients like have a preference about that? Do they ever like say they don’t want to?

S: Sometimes they do, but, um, I have HIV.

Elsa then explained that she was open with her clients about her HIV status but had not experienced any negative responses (enacted stigma):

I: Um, okay, so, um, and when did you find out about your HIV status?

S: Um, probably about a month ago. […]

I: Right. Okay, and it sounds like you feel quite comfortable telling people about your status. It’s not something you hide.

S: No, not at all.

I: Yeah. And how do people react? Are they accepting or are they –

S: Um, most of them, um, seem like they accepted it. Um, there’s been a couple that were – they didn’t even know what to say I guess. […]

I: Yeah, but you haven’t experienced sort of like hostility or anything?

S: No, not yet anyway.

#### Explaining Perceived Changes in Stigma

Interviewees themselves described three key changes to which they attributed what they had observed as a recent reduction in stigma, whether felt or enacted: (1) the availability of effective treatment and reduced threat to health posed by HIV; (2) the inevitability of public knowledge about a person’s HIV status in the small town community of people using drugs and (3) direct personal experience of HIV diagnoses arising from the recent local outbreak.

#### Reduced Threat, Reduced Stigma?

The idea that, for those able to access it, anti-retroviral therapy (ART) has transformed HIV from a disease with extremely high mortality to a manageable chronic illness, is not new (Deeks, Lewin, and Havlir 2013). That this reduced threat to health might account for changing attitudes to HIV corresponds with findings among men who have sex with men using pre-exposure prophylaxis (PrEP) (Koester et al. 2018). PLWH participating in the study mentioned that they were engaged in treatment and several reported having an undetectable viral load. This underserved and impoverished population may even enjoy better health after acquiring HIV than before due to their greater access to health care and other resources. Adam commented “If you catch HIV I believe you get a check and you get a hell of a lot more healthier than you were before. […] A lot of people are looking at it like it's a good thing. It's fucking weird, man.” Staff at the SSP confirmed that patients receive more health care support after diagnosis, including a dedicated health care worker and, if able to meet viral suppression targets, vouchers for food at local grocery stores. Crane et al. observed a similar phenomenon in California describing it as the ‘commodification of AIDS’ in a welfare system where the ‘sick, needy, and addicted must compete against each other for scarce resources’ and in which an HIV diagnosis provides a competitive advantage (Crane, Quirk, and van der Straten 2002). The idea that for some, an HIV diagnosis represents a positive development in their life could explain what participants viewed as a decline in felt and enacted stigma.

Another interpretation of Jenni and Chris’ dismissive attitude towards HIV would focus not on the relative outcomes of health risks faced by PWID or the fear that these risks engender but on attitudes about the behavior that is considered their *cause*. The relatively low levels of stigma around HIV may reflect the in-group acceptability of the injecting behavior behind the local outbreak and a tacit understanding that syringe sharing sometimes occurs. Lisa, a woman in her 30s who had tested positive for hepatitis C but not HIV, confided how she herself had shared syringes, reflecting, ‘You know, just to be completely honest, anybody that does use IV drugs and says they have never shared needles, I would really feel comfortable saying that they’re not telling the truth*.*’

#### Information Transmission and Gossip

Openness about HIV seropositivity was suggested by interviewees’ ease of sharing this information. Interviewers asked about health problems resulting from drug use but did not ask specifically about HIV status. Even so, eight participants volunteered the information that they were living with HIV. Although some of the remaining interviewees may have hidden their diagnosis, it appeared that those who did not volunteer that they were seropositive were HIV negative as it was possible to infer their HIV status based on indirect statements as they discussed hygiene precautions, fears of transmission and acquisition, other friends’ diagnoses, etc. Participants also reported openness about HIV positive status between other PWID and some concealment also. Clarence, a man in his 20 s using heroin for ten years, explained:

I: Um, and are people quite open it or do you find they don’t wanna tell people [about their HIV status]?

S: Some people don’t wanna tell people.

I: Right. Okay.

S: And others do.

I: Yeah.

S: It just depends on who you run into.

Yet this openness did not extend to all interview topics; many chose not to answer questions about their personal sexual activity.

Chris shared the view of several interviewees that keeping one’s HIV seropositivity secret wasn’t feasible given the close, face-to-face community of people using drugs:

If you’re a drug user eventually you’re going to run into other drug users around you. […] Like everybody knows everybody. You can’t hide it [your HIV status]. You can’t hide nothing in here. It’s like having a small knit group of people in a town. […] Eventually you’re going to find out.

As an explanation of tolerance, however, it is problematic, given the many historical examples of close-knit communities that have expelled or persecuted those considered deviant. Perhaps more significant is that Chris sees the spread of information through gossip as an inevitable process, where the fate of individuals is determined by external forces, and he was not alone in this view.

#### Direct Experience of HIV

The experience of HIV recently becoming commonplace among peers who injected drugs may have also have affected perceptions of HIV-related felt and enacted stigma. Andy described how the local experience of HIV had changed his partner’s negative attitudes towards PLWH, recalling, “My, girlfriend – current girlfriend, she, at first, she was a big judger and stuff until, uh, a couple of her close friends, got it. And, uh, I guess it kind of changed her outlook on it.”

Participants were well aware of the mechanisms of transmission through syringe sharing and were making efforts to prevent such transmission, in part evidenced through their attendance at the SSP. However, fatalism about what appeared to some as the inevitable spread of HIV among PWID was common. Adam, in his 30 s and living with HIV, was asked about the recent outbreak in the town and commented, “Everybody has it. Everybody I know. Every fucking person. And if you don't have it, take about five or six tests, you'll have it. Janie, [who you] just talked to—she tested negative five times in a row. Sixth time, positive. It was all like in two months”. This fatalism represented a co-constructed understanding of HIV among PWID that prepared individuals who are HIV negative for the possibility of seroconversion.

It was not always clear the extent to which this perceived reduction in stigma related only to the community of those injecting drugs and how much in the town as a whole. The sense of a boundary between PWID and other members of the town, more typical of enclaves, emerged most strongly among some of the sex workers who were living with HIV. This may have reflected their greater occupational contact with other townspeople. They described stigmatizing behavior and a ‘burn the witch’ attitude from potential clients outside the group of PWID. The sense among the street family of being stigmatized by the outside world for their injection drug use, homelessness and, in some cases, sex work, may also have created a defiance against outside norms, where ‘rejection meets rejection’ (Douglas and Calvez 1990). This defiance of norms can bring a sense of solidarity in adversity and a rejection of stigma imposed from outside the group but these phenomena were not, in themselves, strong enough to form a consistent enclave. These contradictory forces were exemplified in the ‘Hi-V Club’ (pronounced ‘Hi Five’).

### The ‘Hi-V Club’

Accounts of the ‘Hi-V Club’ suggest that enacted stigma continues to be directed from outside the street family but, while inspiring defiance and some sense of shared identity, these are not sufficient to form a bounded enclave that provides for its members. Three of the PLWH mentioned the ‘High Five’ or ‘Hi-V Club’ whose name is a pun that comes from ‘HI-V’ where V = Roman numeral 5 spelling out ‘HIV’. There was disagreement as to whether it was (a) a tight group of friends recently diagnosed with HIV, injecting drugs and living homeless bonded by shared adversity and mutual understanding; (b) PLWH who were members of the street family but with no particular identity *or* (c) merely a trope of shared dark humor. One of the members declined to be quoted directly but expressed the view that only people within the High Five Club really understood the experience of living with HIV and Chris observed that he felt closer to those with HIV than to others.

Sylvie gave a different interpretation, explaining ‘It’s a joke for all of us around [the town] that we’re new, we’re all heroin shooters, we’re junkies, that’s all we do. Um, and we’re all—we’ve all—have tested HIV positive in the last, you know, six months, year.’ Further questioning went as follows:

I: […] Is there something that binds you together more than just like a joke or –

Sylvie:Um, I wish that I could say ‘yes’ because there should be […] but no, there’s not, no, we don’t all share anything or share, you know, a special bond.

Either way, its ingenious name exemplified a defiant attitude towards HIV-related stigmatization. Sylvie describes here a reaction to their reported pariah status in the town, drawing a boundary around themselves against mainstream society: ‘So that is our bonds that, you know, well everybody hates us and whatever, you know, that’s like, you know, we’ll never—“You’ll never have to deal with it alone, ha, ha, ha, you know […] You’ve got your friends in the Hi-V Club.”’ The use of humor and irony as a coping mechanisms or form of deflection for stigmatized status has also been observed in other contexts (Jensen 2018; Zilberg 1995; Black 2012).

Like the wider street family from which its members were drawn, the Hi-V Club showed aspects of both enclaves and isolates in which behavior as a bounded group was inconsistent. The culture of isolates tends towards blaming external forces for adversities, whether other individuals or groups. A particularly vivid example of this is in the conspiracy theories that were circulating about the origins of the recent HIV outbreak. Such theories speak to a fatalistic lack of control over events and a sense of being the victims of others’ actions. These conspiracy theories can be dismissed with psychological explanations of mental illness or a lack of evidential knowledge but they are often found among isolates and dissenting enclaves. In dissenting enclaves they may be unified and consistent while among isolates they may vary more widely (Calvez 1995).

As explanations of causation, the conspiracy theories attributed responsibility or blame for the local HIV outbreak to the recent appearance of higher potency methamphetamine in a combination of supernatural and government forces bringing about the end days of the Apocalypse; to a group of established townspeople who wished to rid the town of an undesirable element by deliberate infection with HIV and, finally, to the local needle and syringe program putting HIV into the syringes. This last was reported third hand as heard from others rather than held by the interviewee himself.

While a cultural bias towards fatalism can impede the ability to organize and provide mutual support as a group, it may also allow individuals to place less responsibility for adversity on themselves and hence reduce blame and enacted and felt stigma. If PWID see themselves as victims rather than responsible for viral transmission, they may be more accepting of each other. Along with the other factors such as advances in medication, acceptance of injecting and the occurrence of equipment sharing, and the many other immediate health threats facing people living homeless and using drugs, fatalism may help explain the perceived reduction in stigma described by members of the street family during the recent outbreak.

### Limitations

While the data is remarkably rich and many of the interviewees shared intimate details of their personal lives, it was gathered during a single week of research and represents only a snapshot. One interview was carried out per person and comparison with some of the parallel HIT study’s data suggests that interviewees may have been more open to confiding in the interviewers during second interviews or during ethnographic work. Future studies could include serial interviewing or the addition of ethnography.

Interviews seem to be a useful way to capture data on beliefs, perceptions and past experiences but for behavior, which needs to be observed, ethnography is needed (Fessel et al. 2019). Participants often chose not to discuss their own sex lives and this limited our understanding of sexual risk. There was also potential for social desirability bias resulting from the institutional setting of the NSP but the interviewers tried to overcome these by asking similar questions in different ways. With all qualitative research and particularly studies carried out among hidden populations, it is unknown how representative findings are of their wider population. Further research is needed to gain an understanding of whether similar developments are occurring on a broader scale.

### Discussion

There was strong agreement that within the street family and among others who injected drugs that the experience of felt and enacted HIV-related stigma had greatly diminished in the wake of the recent outbreak. Some possible causes were observed by the participants including the reduced threat to life HIV represents when controlled by ART, the prospects of improved health *after* diagnosis and the commonplace incidence of HIV among friends and acquaintances. A Cultural Theory analysis suggests that fatalism arising from the living situations and cultural characteristics of the street family may also play a part.

Cultural Theory analysis draws our attention to the social forces by which people are organized or resist organization and the effects these have on perceptions of autonomy and the control of risks, on perceived timespans and other cultural practices, values and beliefs. Marginalization by mainstream society, its proponents argue, can create the conditions for solidarity in shared adversity and defiance of wider social expectations around HIV-related risk (Douglas and Calvez 1990). This could also explain the rejection of HIV-related stigma among the street family who are already marginalized by injecting drug use, homelessness, separation from family ties and in some cases, sex work. Yet although the pull towards unity existed, the daily short-term demands of maintaining a personal opiate supply and other essentials while living in extreme poverty dominated decision-making, fragmenting the fragile bonds that existed between those living homeless. In this way, drug dependence and poverty acted as a ‘grid’ of its own—a set of internal rules that took precedence over longer term considerations. Understanding the isolate characteristics (high grid, low group) of the ‘street family’ and the Hi-V Club allows us to see how immediate needs undermine their aspirations to forming a mutually supportive group, and yet also contribute to mutual tolerance and acceptance.

It could be argued that the lack of organized structures and norms found among the street family can be attributed to the relative newness of injected heroin/fentanyl use and HIV in West Virginia’s street scenes (Ondocsin et al. 2020). Some ethnographic research among more established urban drug scenes in the US shows groups of PWID with a greater sense of shared rules and norms (Page et al. 1990) but these do not inevitably develop over time. Economic, political and social forces help to shape the different solidarities that emerge in different contexts and their degree of ‘grid’ and ‘group’. The divisive effects of economic pressures on people using heroin in New York City’s street scene in the 1960s led to the likelihood of their robbing each other for drugs or money, a practice also reportedly common among members of the WV street family (Preble and Casey 1969). A study of Puerto Rican PWID in both Connecticut and Puerto Rico found that their high risk behavior for HIV transmission resulted in part from prolonged histories of oppression and marginalization (Singer 1999).

‘HIV fatalism’ has been noted in studies in sub-Saharan Africa in relation to HIV risk taking and health promotion (Sileo et al. 2019; Hess and McKinney 2007). However, these tend to consider fatalism as a psychological trait rather than a form of explanatory framework, and sometimes attribute to it a total approach in which everything in a person’s life is considered to be controlled by external forces. While elements of fatalism are evident in this study, particularly among the street family, it emerged in a more nuanced form. For instance, participants reported engaging in efforts to prevent HIV through use of sterile injecting equipment but the outcomes of these efforts were not considered entirely explainable through personal agency. Fatalism here takes the form of a co-constructed understanding of HIV among members of the street family that prepares individuals who are HIV negative for the possibility of seroconversion, where HIV’s ‘just another thing’ and which may limit effort to reduce risk of HIV exposure.

As we have observed more recently with COVID-19 in the US, when there are great inequalities of wealth, the effects of pandemics tend to fall harder upon the marginalized and the urban poor than where wealth is more evenly distributed (Risk and Blame 1992). Similarly, the HIV epidemic has become a disease of poverty and marginalization. The US is the most unequal developed western nation and, among its states, West Virginia is one of the poorest. Reduced stigma within a marginalized group will not improve acceptance and incorporation by mainstream society. The openness that could reduce HIV transmission risk will not be sufficient to prevent outbreaks if there is no long-term prospect of a better life in the future. The decline in stigma, though encouraging, is small comfort when the conditions of poverty and marginalization which create greater risk of HIV acquisition and transmission persist.
